# Prevalence and clinical characteristics of Crooke’s cell adenomas in 101 patients with T-PIT-positive pituitary adenomas: Case series and literature review

**DOI:** 10.3389/fendo.2022.947085

**Published:** 2022-08-19

**Authors:** Dimin Zhu, Zongming Wang, Tian Tian, Xinyi Wu, Dongsheng He, Yonghong Zhu, Dawei Liu, Haijun Wang

**Affiliations:** ^1^ Department of Neurosurgery and Pituitary Tumor Center, The First Affiliated Hospital of Sun Yat-sen University, Guangzhou, China; ^2^ Department of Pathology, The First Affiliated Hospital of Sun Yat-sen University, Guangzhou, China; ^3^ Department of Histology and Embryology, Zhongshan School of Medicine, Sun Yat-sen University, Guangzhou, China

**Keywords:** Crooke’s cell, pituitary adenoma, ACTH, Cushing disease, hyalinization

## Abstract

**Purpose:**

We aimed to perform a retrospective analysis of a rare subtype of corticotroph adenoma, Crooke’s cell adenoma, to better understand its clinical features.

**Methods:**

We collected *T-PIT*-positive pituitary adenomas and screened Crooke’s cell adenomas from January 2020 to December 2021 in our center. Case reports of such tumors were also collected through a literature search. Clinical data such as biochemical tests, imaging examinations, and pathological data of the above cases were analyzed.

**Results:**

A total of 101 *T-PIT*-positive patients were treated in our center in the last 2 years, and 4 were finally pathologically diagnosed with Crooke’s cell adenomas. All of these patients were male with elevated adrenocorticotropic hormone levels, and 50.0% presented with hypercortisolemia, Cushing’s syndrome, visual impairment, and headache. The tumor diameter was significantly larger in these 4 patients (37.0 mm) than in the other patients (26.0 mm), and their tumor invasive behavior was more pronounced. Cases reported in the literature were mainly female (72.8%), and the clinical presentation was also dominated by Cushing’s syndrome (65.1%) and hormonal dysfunction. Tumors were more common as macroadenomas (33.2 mm) and suprasellar growths (63.8%). The tumor recurrence rate was as high as 55.6%, with 6 cases progressing to pituitary carcinomas and 7.7% of tumor-related deaths. Our further integrated analysis of our center and reported cases revealed that gender, Cushing’s syndrome, visual dysfunction, hormonal disorders, and tumor growth characteristics were statistically different in different tumor categories.

**Conclusion:**

Crooke’s cell adenoma is a tumor subtype with obvious clinical aggressive behavior, and an in-depth analysis of its clinical characteristics may assist in developing a comprehensive treatment plan.

## Introduction

Pituitary Crooke’s cell adenomas (CCAs) are a rare subtype of corticotroph adenomas typically associated with Cushing’s disease, accounting for less than 1% of pituitary adenomas (1). These tumors, first described by Arthur Carleton Crooke in 1935 (2), represent a distinct clinicopathological subtype of pituitary adenomas. They were remarkably aggressive and showed the characteristics of Crooke’s cell - cytokeratin (CK) filaments accumulating heavily around the nucleus, making them appear distinctly hyaline in hematoxylin and eosin (HE) staining. The neoplastic Crooke’s cells are strongly immunoreactive *T-PIT* and *CK8/18* and exhibit variable adrenocorticotropic hormone (*ACTH*) immunoreactivity. It has been suggested that CCA should meet the diagnostic criteria of at least 50% of neoplastic Crooke’s cells in corticotroph adenomas. The presence of hyaline change is reported to be the result of respondence of excess glucocorticoids, but the mechanisms have not been well understood. CCAs usually take the form of invasive macroadenomas with a high rate of recurrence after standard resection. We reviewed 4 cases of CCA, with clinical, radiological, and histopathological features. We are seeking a better understanding of their clinical-pathological characteristics, as well as assessing their immunophenotype and prognosis.

## Materials and methods

### Patient information

We performed a retrospective analysis of patients with pituitary adenoma who underwent surgery at The First Affiliated Hospital of Sun Yat-sen University from January 2020 to December 2021. All masses were removed by the same surgical team, and their pathological findings supported the diagnosis of *T-PIT*-positive pituitary adenoma. We identified CCAs with a multidisciplinary synergistic diagnosis of neurosurgery, pathology, endocrinology, etc.

### Biochemical and imaging data

During the perioperative period, hormonal and imaging studies are performed in our center on patients with pituitary adenomas. The reference ranges for hormones are as follows: 8 AM Cortisol, Urinary cortisol, 24h urinary cortisol, *ACTH*, *PRL*, *GH*, *TSH*, *LH*, and *FSH*. Above or below these ranges are considered abnormal hormone secretion. We used 3.0T Magnetic Resonance Imaging (MRI) for routine tumor screening. For the pituitary gland, thin cuts in coronal and sagittal positions are used to facilitate visualization of the cavernous sinus and optic chiasma. For microadenomas, dynamic MRI scans have been used to increase the sensitivity; while for macroadenomas, conventional coronal and sagittal pituitary MRI with contrast is generally adequate for treatment planning. The direction of tumor growth was classified as supra-sella, para-sella (cavernous sinus), clivus and infra-sella (sphenoidal sinus).

### Pathological data

We used HE staining and reticulin fiber staining for the initial diagnosis of tumorigenic lesions in the sella area masses, based on which *T-PIT* (AM0486), *PIT-1* (AM0451), and *SF-1* (AM0443) immunostaining was used to distinguish the tumor cell spectrum origin. The transcription factor antibodies are all purchased from Xiamen Talent Biomedical Technology Co., Ltd. (Fujian Province, China). *ACTH*, *PRL*, *GH*, *TSH*, *LH*, *FSH*, and *CK8/18* immunostaining was used for further classification of tumor categories. The *Ki-67* index was used to determine the proliferative activity of the tumor. The final pathology report was issued by two experienced pathologists after discussion.

### Review of the literature

We searched the PubMed database using the terms “Crooke’s cell”, “Crooke’s cell hyaline deposition”, and “Crooke’s cell adenoma”, etc. We reviewed all relevant English-language literature published before December 2021 and performed a summary analysis of the case reports.

### Statistical analysis

Statistical analysis was performed using SPSS software (version 19.0, IBM Corp.). Continuous variables with normal distribution were presented as mean ± standard deviation. Comparing two sets of quantitative data following a normal distribution using the Student’s t test. The frequencies of categorical variables were compared using a chi-square test. A value of *p*<0.05 was considered statistically significant.

## Results

### Prevalence of CCAs

Over the past 2 years, we treated a total of 418 patients with pituitary-related disorders, of whom 391 completed pathological testing for transcription factors and hormones, while a total of 101 patients (25.8%) were positive for *T-PIT*. 4 patients eventually received a pathological diagnosis of CCA, the prevalence of which was approximately 1.0%.

### Characteristics of CCAs

Of the 391 patients, there were 244 clinically silent adenomas, of which 66 (27.0%) were *T-PIT* positive cases. As shown in **Table 1**, of the 97 non-CCA cases recorded in our center, their average age was 45.5 ± 14.0 years, and 17.5% (17/97) were male; whereas no female patients were found in CCA cases and the mean age was 45.0 ± 8.0 years. Clinically, a total of 18 (18.6%) non-CCA patients with significant clinical symptoms associated with Cushing’s Syndrome, and 50.0% (2/4) CCA patients exhibited similar symptoms. Visual dysfunction was observed in 50.5% (49/97) of non-CCA patients and 50.0% (2/4) of CCA patients. 25.8% (25/97) of non-CCA patients and 50.0% (2/4) of CCA patients presented with headache. For hormone secretion, 8.3% (8/97) of non-CCA patients had hypercortisolism, while half of the CCA patients had significantly elevated cortisol levels. All CCA cases had abnormal secretion of *ACTH*, while non-CCA patients presented more often with non-functioning adenomas (NFPAs), with only 18.6% (18/97) having this hematological feature. The overlap with clinical and hormone immunohistochemistry in 101 cases was shown in **Supplemental Table 1**. The mean tumor diameter was 26.0 mm in non-CCA patients and 37.0 mm in CCA patients, the latter being significantly larger than the former. Tumors in CCA patients showed significant invasive behavior to surrounding structures such as the cavernous sinus (100.0%, 4/4), sphenoidal sinus (100.0%, 4/4), suprasellar region (75.0%, 3/4), and posterior cranial fossa (75.0%, 3/4). Non-CCA tumor invaded mainly towards the suprasellar region (59.8%, 58/97) and the cavernous sinus (50.5%, 49/97). The recurrence rate of CCA (75.0%, 3/4) is significantly higher than that of non-CCA (39.2%, 38/97). One CCA patient (25.0%, 1/4) died due to tumor complications.

**Table 1:** Characteristics of our cases.

Classification			Ratio (%)	
			CCA (n=4)	non-CCA (n=97)
Gender	Male		100.0 (4/4)	17.5 (17/97)
	Female		0	82.5 (80/97)
Age (y)			45.0 ± 8.0	45.5 ± 14.0
Clinical manifestation	Cushing’s syndrome		50.0 (2/4)	18.6 (18/97)
	Visual defect		50.0 (2/4)	50.5 (49/97)
	Headache		50.0 (2/4)	25.8 (25/97)
Hormonal dysfunction	Hypercortisolemia		50.0 (2/4)	8.3 (8/97)
	Increased ACTH		100.0 (4/4)	18.6 (18/97)
Biological characteristics of tumor	Diameter (mm)		37.0 ± 16.5	26.0 ± 12.7
	Growth direction	Suprasellar	75.0 (3/4)	59.8 (58/97)
		Cavernous sinus	100.0 (4/4)	50.5 (49/97)
		Sphenoid sinus	100.0 (4/4)	35.1 (34/97)
		Posterior fossa	75.0 (3/4)	16.5 (16/97)
	Recurrence		75.0 (3/4)	39.2 (38/97)
	Tumor Related Death		25.0 (1/4)	0

### Cases presentation

#### Case 1

**Figure 1:**
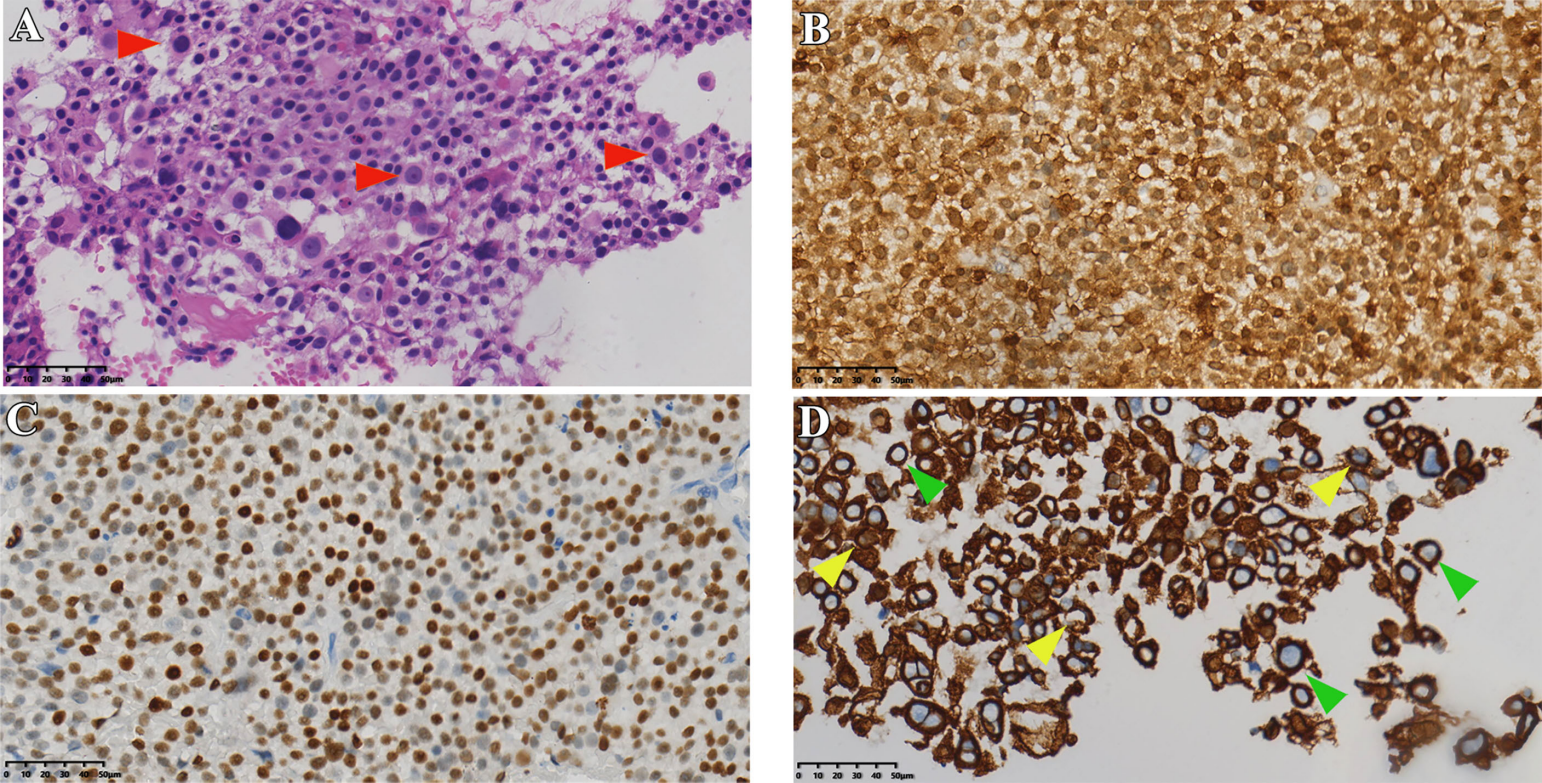
Histopathological data of case 1. **(A)** HE staining of tumor tissue (red arrows indicate typical Crooke’s hyaline change). **(B, C)** Immunohistochemical staining for *ACTH* and *T-PIT*. **(D)** Immunohistochemical staining of cells keratin showing Crooke hyaline changes (green and yellow arrows show a round and semi-circular immunostaining of *CK8/18*, respectively). Original magnification, 40×.

**Figure 2:**
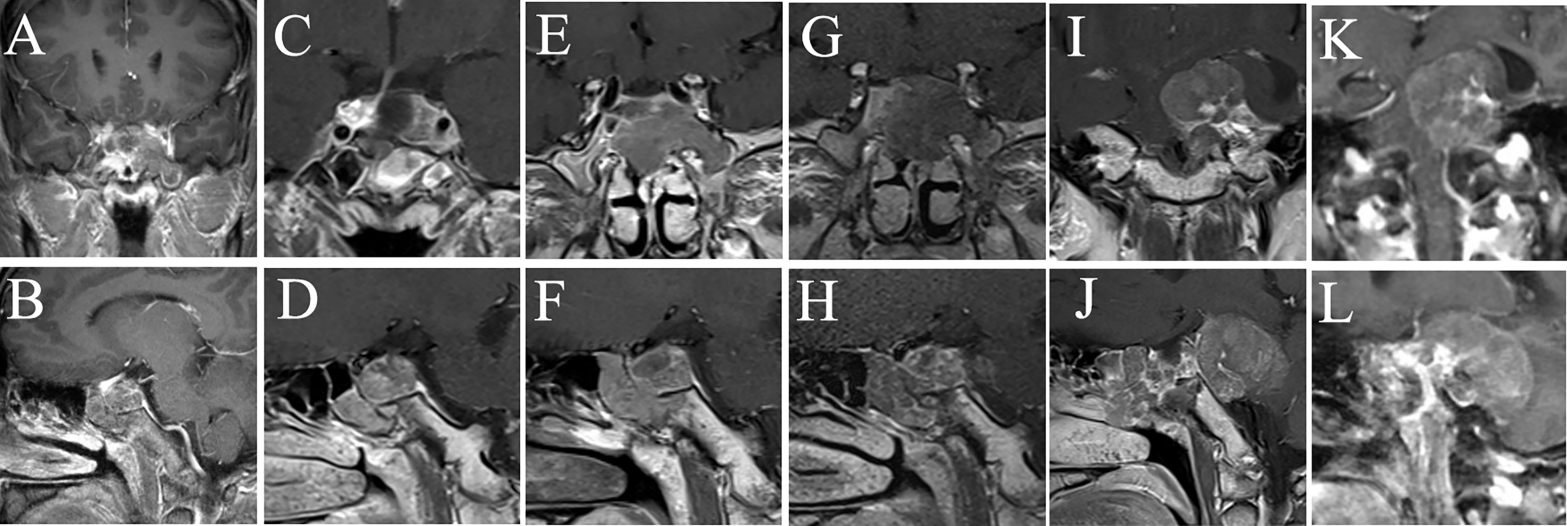
MRI examination of case 1. The images show the patient’s coronal and sagittal MRI of the head before **(A, B)**, one week after **(C, D)**, six months after **(E, F)**, eight months after **(G, H)**, sixteen months after **(I, J)**, and seventeen months after **(K, L)** the fourth surgery, respectively.

A patient aged 32 suddenly developed symptoms such as right eyelid ptosis and visual impairment in 2016, and cranial MRI suggested a 18×38×30 mm (Knosp grading 4) lesion in the sella area. Transsphenoidal resection was performed in November of the same year, and postoperative pathology confirmed the *ACTH* adenoma. Clinical symptoms related to the occupying effect of the tumor were alleviated, but the MRI suggested tumor recurrence in the second month after the operation. Transsphenoidal surgery was performed again, followed by gamma knife radiotherapy while the MRI showed a residual tumor in the left cavernous sinus area. In February 2018, the patient presented with weakness, memory loss, abdominal purple striae, and central obesity. Cranial MRI suggested a 18×19×9 mm lesion in the sella area. In September 2018, after intolerable side effects from mifepristone, he underwent bilateral adrenalectomy in our hospital and gradually developed postoperative hyperpigmentation of the face and limbs. In December, nasal bleeding was appeared and endoscopy revealed a neoplasm at the olfactory fissure protruding into the common nasal tract with a maximum diameter of about 34 mm, and tumor recurrence is considered. In January 2019, a surgical resection was performed and the pathology reported an aggressive *ACTH* adenoma with *Ki-67* of about 20%. A residual lesion was found and radiotherapy was re-performed postoperatively. In July 2019, symptoms of nasal bleeding re-emerged and *ACTH* was significantly elevated (**Table 2**). A nasal endoscopic biopsy was performed and the pathology suggested CCA (**Figure 1**). In April 2020, the pituitary MRI showed a recurrent mass of 37×43×34 mm in the operated area encapsulating the left internal carotid artery (**Figure 2**). In August 2020, the mass had invaded into the cerebellopontine angle region, and the headache was relieved after surgery, but vision loss and left-sided facial hypoesthesia occurred. In November, MRI scanning showed that the mass size increased to 53×39×26 mm, compressing the left temporal lobe and brainstem. After considering the risk of surgery, the patient and his family decided to take “Temozolomide 100 mg/day combined with Anlotinib 8 mg/day”. At the same time, symptomatic treatments such as hormone replacement, intracranial pressure reduction, and maintenance of water-electrolyte balance were administered. The patient was discharged from the hospital with occasional symptoms such as choking on water, difficulty urinating, chest tightness, dizziness and headache, which were treated symptomatically. MRI was repeated in December 2020, and the lesion further expanded to 65×47×50 mm, involving the temporal lobe and brainstem, with supratentorial hydrocephalus. Unfortunately, the patient died in 2021 due to severe complications.

**Table 2:** Hormonal changes in displayed cases.

Hormone	Case 1		Case 2		Case 3		Case 4	
	Pre	Post	Pre	Post	Pre	Post	Pre	Post
Cortisol (8 AM, 2.90-19.40μg/dL)	1.00↓	15.70	28.60↑	11.80	19.70↑	13.40	12.30	19.20
Urinary cortisol (0.43-11.70μg/dL)	82.50↑		29.10↑	38.20↑	4.00			
24h urinary cortisol (4.30-176.00μg)	3300.00↑		742.05↑	1356.10↑	112.00			
ACTH (1.60-13.90poml/L)	>440.40↑	159.2↑	41.91↑	7.81	22.27↑	9.67	17.72↑	9.58
PRL (1.61-18.77ng/mL for male)	14.49	<0.60↓	17.68	4.31	12.67	6.72	19.25↑	7.69
GH (0-10ug/L)	0.11	0.11	0.31		0.18	0.72	0.69	
TSH (0.56-5.91uIU/mL)	1.85	0.4↓	0.28↓	0.37↓	1.75	0.91	2.20	0.61
LH (2-12IU/L)	2.57	1.95↓	2.36	3.27	1.55↓	5.90	5.71	4.69
FSH (1-8IU/L)	4.51	3.14	7.12	5.07	5.57	8.15↑	9.49↑	9.98↑

Pre, pre-operative; Post, post-operative; ↑ means elevated; ↓ means reduced.

#### Case 2

**Figure 3:**
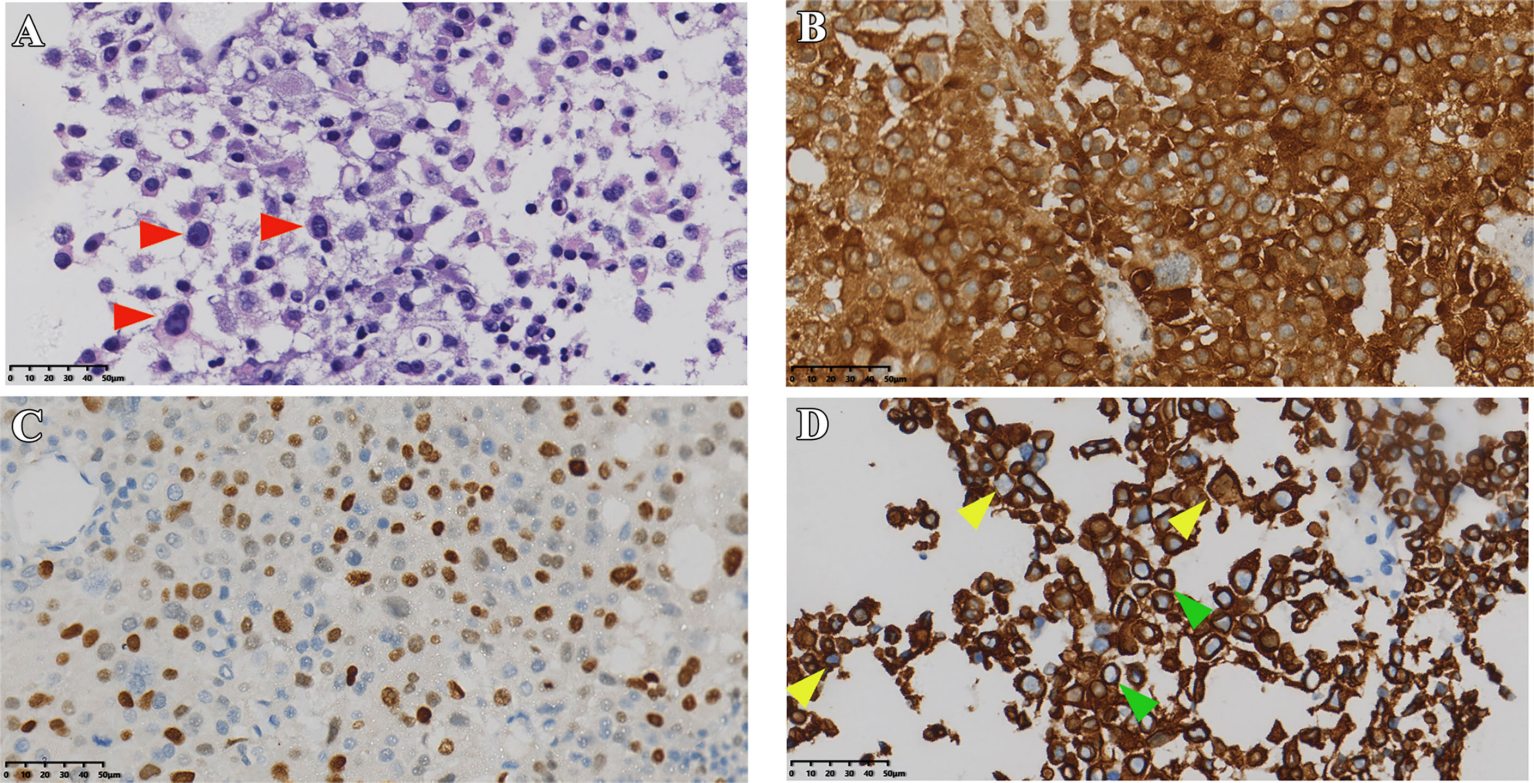
Pathological sections of case 2. **(A)** HE staining of tumor (red arrows indicate typical Crooke’s hyaline change). **(B, C)** Immunostaining of *ACTH*, *T-PIT*, and *CK8/18* **(D)** green and yellow arrows show a round and semi-circular immunostaining of keratin, respectively). Original magnification, 40×.

**Figure 4:**
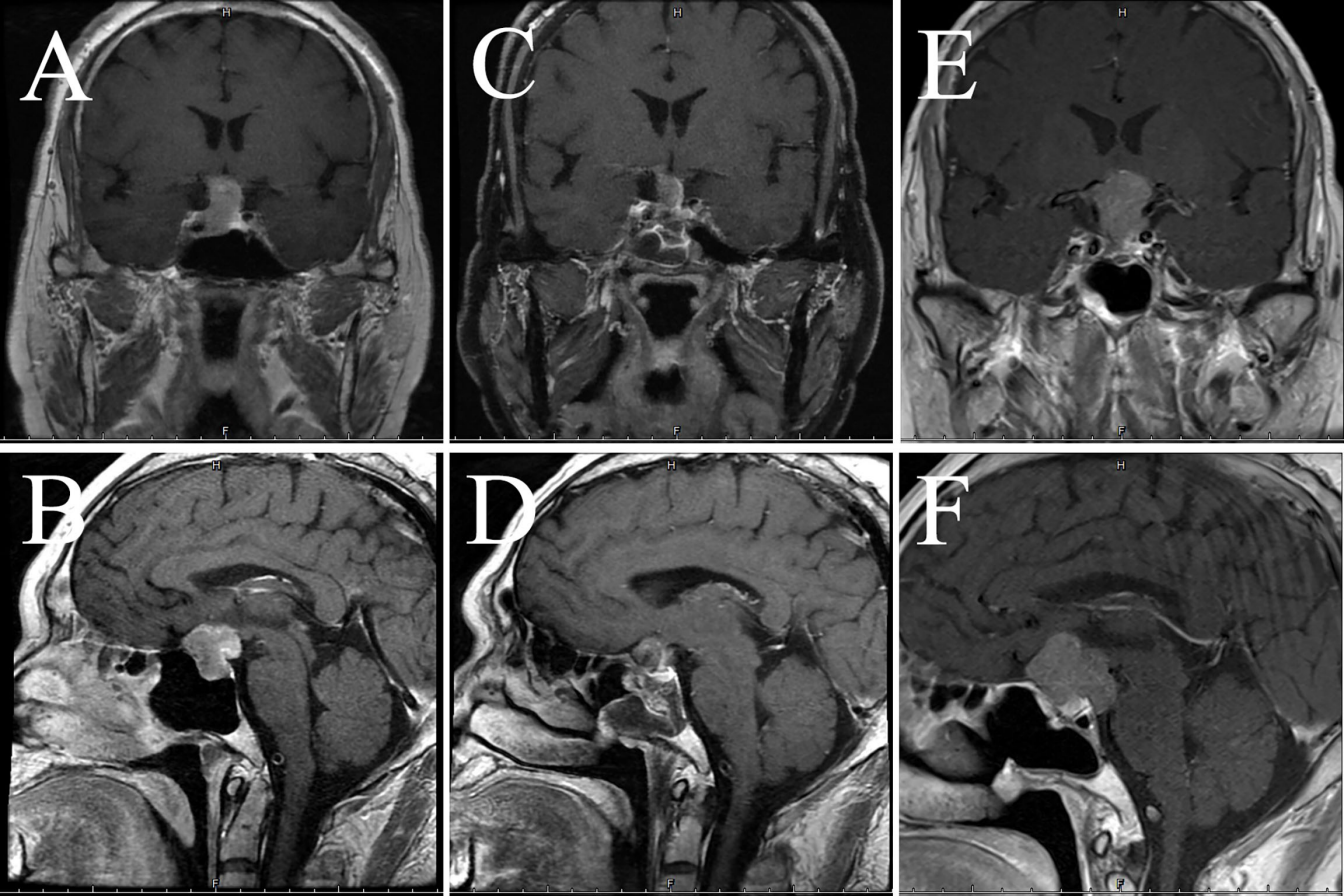
Imaging data of case 2. The images show coronal and sagittal MRI of the patient’s head preoperatively **(A, B)**, three months postoperatively **(C, D)**, and twenty-one months postoperatively **(E, F)**, respectively.

A 54 years old male patient gradually developed symptoms such as hyperphagia, full moon face and buffalo back 7 years ago, without accompanying purple striae, subcutaneous bleeding spots or petechiae. Starting in 2017, he developed generalized skin pigmentation, which was evident in the axillae, buttocks, neck, and joints of the extremities, accompanied by swelling of both lower extremities. In June 2020, the patient was found to have elevated blood pressure, significantly elevated blood cortisol and *ACTH*. MRI of the pituitary gland suggested an occupying lesion of about 25×16×24 mm (Knosp grading 2) in the sella area, with compression of structures like optical chiasma and cavernous sinus. Initial consideration was given to pituitary macroadenoma. Abdominal CT showed multiple nodular hypodense shadows in the left adrenal gland. After admission to our hospital, *ACTH*, cortisol and 24-hour urinary cortisol were found to be elevated (**Table 2**). The patient underwent transsphenoidal surgery in July 2020, and the postoperative pathology suggested a CCA with *CK8/18* > 50% (**Figure 3**), *T-PIT* (+), and *Ki-67* about 3%. No significant postoperative urinary cortisol relief was seen, and no evident residual mass was found in the second day postoperative follow-up CT. However, MRI after 4 months suggested a mass in the operative area, measuring approximately 15×18×14 mm (**Figure 4**). Cortisol concentration was still high at 34 ug/dL at 8 months after the intervention. Twenty-one months after surgery, blood cortisol was 32.1 ug/dL, ACTH was 59.79 pmol/L, and MRI showed a 22*29*23 mm mass in the sellar area. The patient underwent a second surgery in our hospital in April 2022. Intraoperatively, the tumor was seen to be separated by the pseudocapsule, with a tough texture, and pathological staining showed Ki-67 8%. Cortisol and ACTH did not return to normal on repeat examination, and CT suggested residual tumor.

#### Case 3

**Figure 5:**
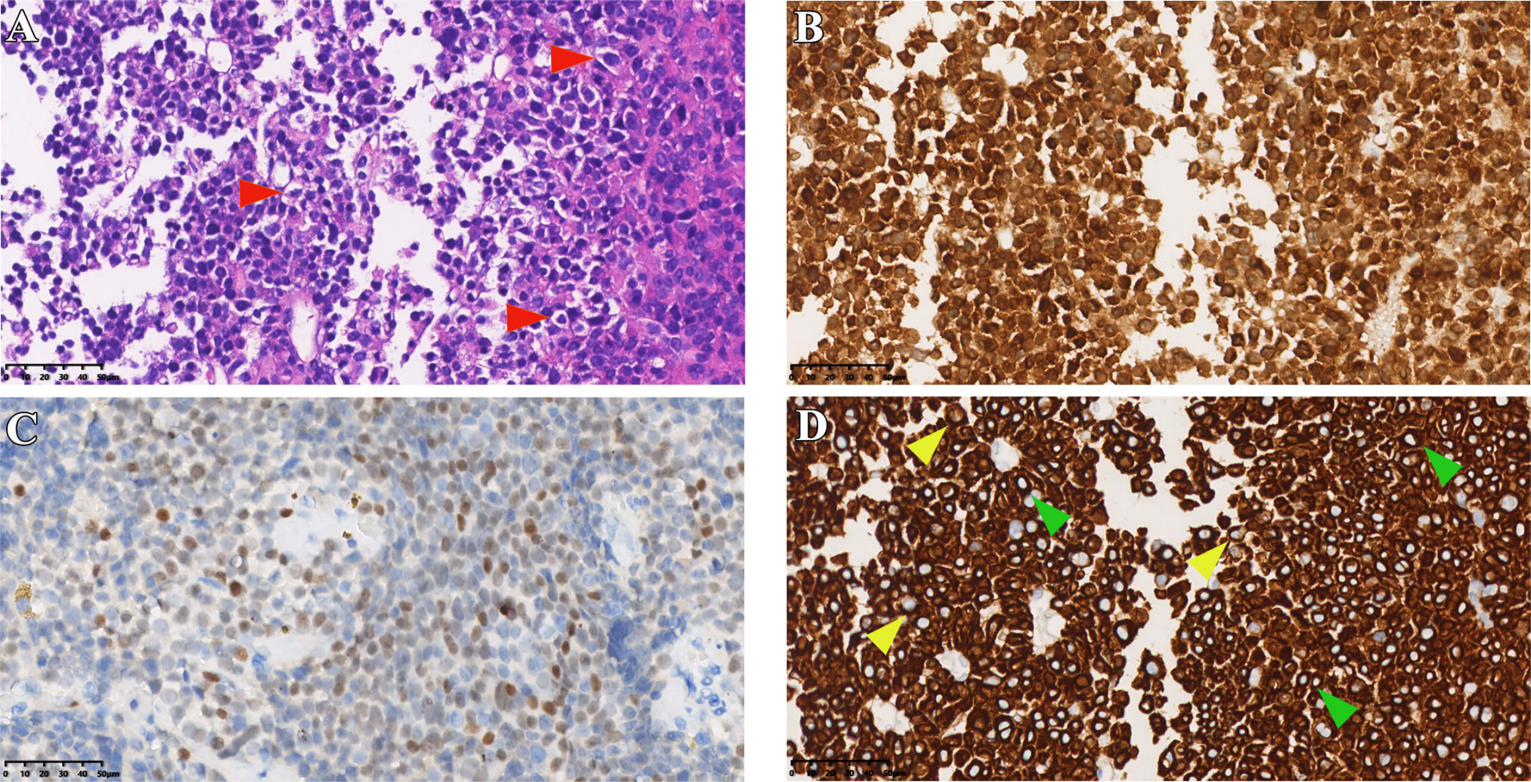
Immunostaining results of case 3. **(A)** HE staining of tumor tissue (red arrows indicate typical Crooke’s hyaline change). **(B, C)** Immunohistochemical staining for *ACTH* and *T-PIT*. **(D)** Immunohistochemical staining of cells keratin (green and yellow arrows show a round and semi-circular immunostaining of *CK8/18*, respectively). Original magnification, 40×.

**Figure 6:**
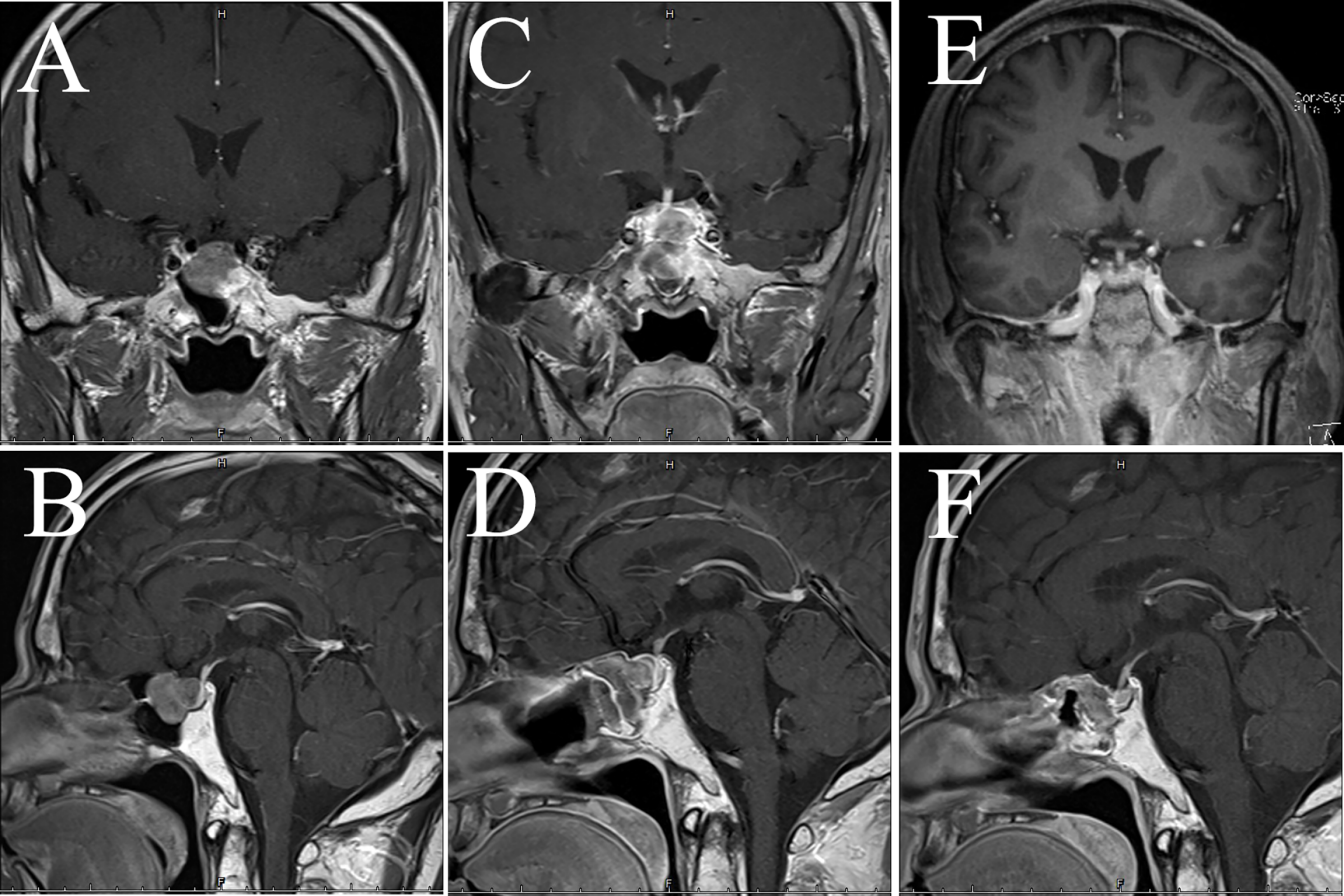
MRI examination of case 3. The images show coronal and sagittal MRI of the patient’s head preoperatively **(A, B)**, one week postoperatively **(C, D)**, and three months postoperatively **(E, F)**, respectively.

One man presented with dizziness, occasional nausea and vomiting at the age of 42 in 2018. Starting the following year, the above symptoms got worse, so he went to our outpatient clinic in 2021. Pituitary MRI suggested an enlarged pituitary fossa and an abnormal signal occupying the lesion in the sella, the size was about 24×20×17 mm (Knosp grading 3), the initial consideration was a pituitary macroadenoma. 1 month later, the head CT revealed that the mass diameters were about 25mm, 23mm, and 18mm. The tumor was aggressive and the bone of the sella base was damaged, and the cavernous sinus was also compressed. The patient underwent microsurgery in October 2021, following which the elevated *ACTH* was relieved (**Table 2**), and the pathology suggested cyclic *CK8/18* staining of about 80% (**Figure 5**), *Ki-67* of about 10%, and infiltration of Crooke cells were observed in bone trabeculae. Hematology and MRI in January 2022 showed no evidence of recurrence (**Figure 6**).

#### Case 4

**Figure 7:**
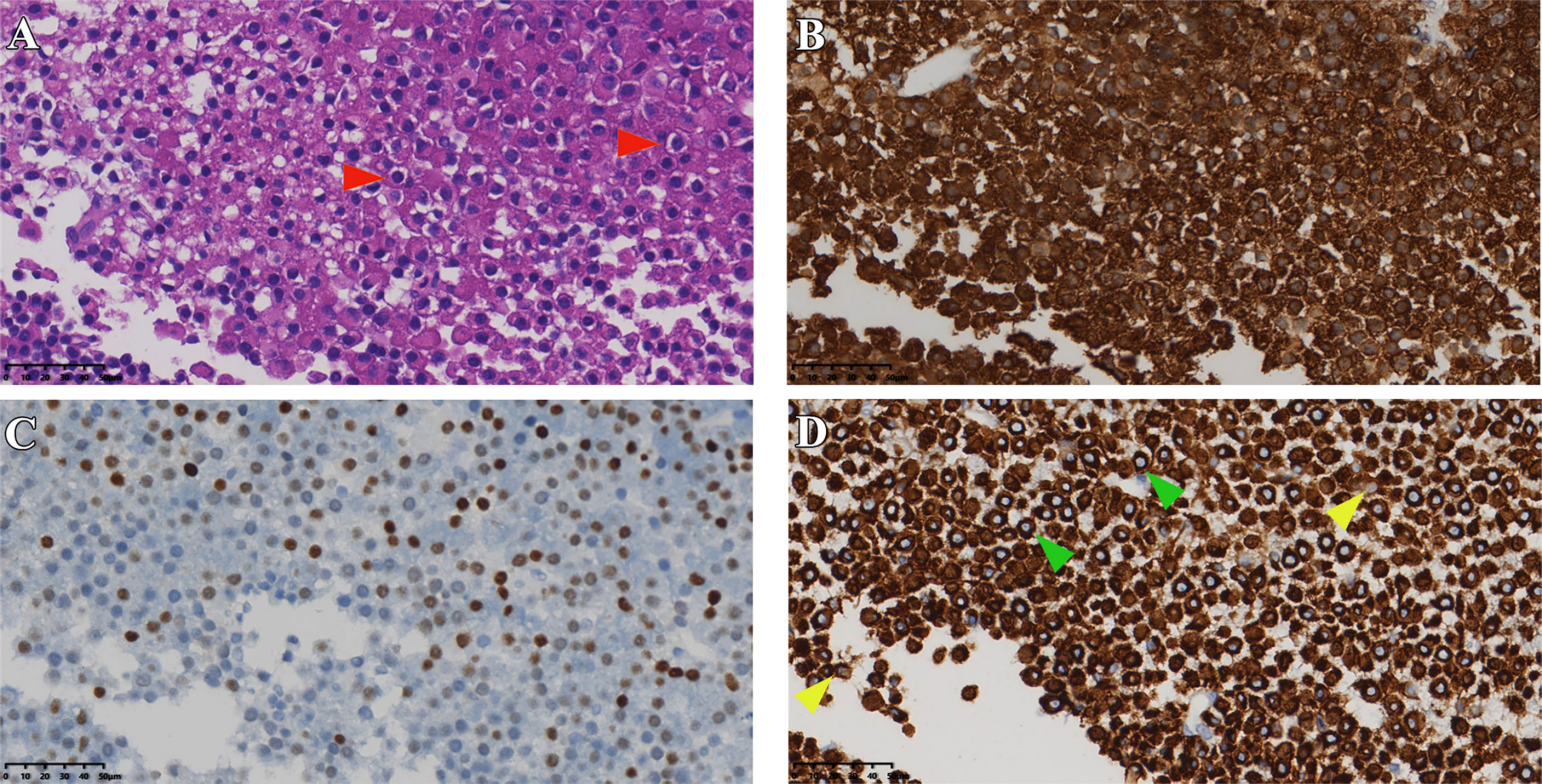
Histopathological staining of case 4. **(A)** HE staining of tumor tissue (red arrows indicate typical Crooke’s hyaline change). **(B, C)** Immunohistochemical staining for *ACTH* and *T-PIT*. **(D)** Immunostaining of *CK8/18* (green and yellow arrows show a round and semi-circular pattern, respectively). Original magnification, 40×.

**Figure 8:**
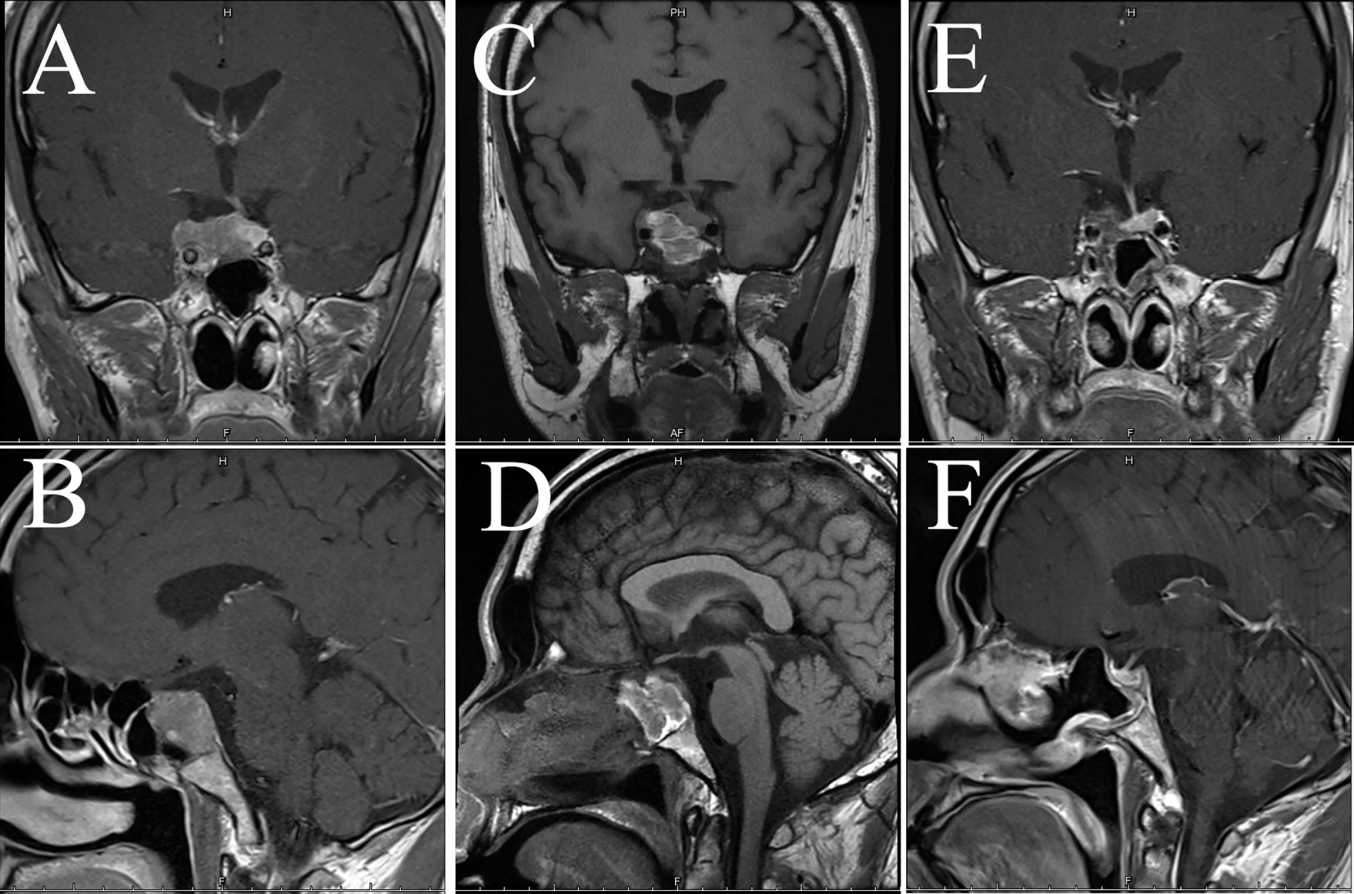
Imaging examination of case 4. The images show coronal and sagittal MRI of the patient’s head preoperatively **(A, B)**, one week postoperatively **(C, D)**, and three months postoperatively **(E, F)**, respectively.

Case 4 is a 50-year-old man with a headache following a “resection of left vocal cord squamous cell carcinoma” in December 2021. The headache was mainly in the right temporal region, with paroxysmal traction pain, with double vision, blurred vision and drooping right eyelid, and elevated *ACTH* on blood examination (**Table 2**). The cranial MRI showed an occupancy in the sella area with a size of about 21×17 mm (Knosp grading 3), and a pituitary macroadenoma was considered. The head CT showed an irregular mass in the sella and supra-sella area, about 33×22 mm in size, with uniform density. The mass wrapped around the siphon segment of the right internal carotid artery and part of the left posterior communicating artery. Also, the neoplasm was close to the left internal carotid artery, and invaded the dorsum of the sella and part of the slope bone downward. The magnetic resonance DTI sequence showed that part of the right optic nerve fiber bundle was interrupted and significantly reduced compared to the other side. Transsphenoidal surgery was performed, and the pathology suggested that the tumor tissue was *T-PIT* (+), *ACTH* positive >95%, Crooke cells accounted for about 80% (**Figure 7**), and *Ki-67* about 1% (+). Normalized *ACTH* was found in a postoperative reexamination. However, recent imaging data suggested a suspicious residual tumor (**Figure 8**).

### Literature review and statistical analysis

To date, over 100 CCA cases have been published (**Table 3**) (3–35). Since the hyalinization of corticotropin cells was proposed by Crooke in 1935 (2), Felix et al. (3) first reported 3 cases of adrenocorticotropin adenoma with a large amount of Crook’s hyaline deposition in 1981. Since then, cases of a variety of sizes have come forward. In 2003, George et al. reported the largest number of 36 cases to date (13). The authors described their clinical manifestations, pathological manifestations, and therapeutic strategies, and underlined their invasive clinical features. Early symptoms in some cases were consistent with asymptomatic NFPAs. With the development of the disease, they are transformed into functional tumors of hypercortisolism, and finally into pituitary cancer. CCA is mainly macroadenoma with obvious clinical invasiveness. In comparison to microadenomas, the macroadenomas that cause Cushing’s disease have different biochemical characteristics, invasive nature, initial remission rate, and post-operative recurrence rate.

**Table 3:** Literature summary of Crooke’s cell adenomas.

Cases no.	Gender	Age (y)	Clinical Manifestation	Hormonal Dysfunction	Mean Diameter (mm)	Invasive Direction	Refractory Cases	Metastases	Management	Tumor Related Death	Ref.
3	3F	36	100% (3/3) CS	100% (3/3) HC and EA	NM	NM	NM	NM	ST	NM	Felix et al. (1981) (3)
7	NM	NM	NM	NM	NM	NM	NM	NM	NM	NM	Martin et al. (1982) (4)
1	F	56	CS, HA	HC	17	SS	no	no	ST	no	Horvath et al. (1983) (5)
10	NM	NM	CS	NM	NM	NM	NM	NM	NM	NM	Robert et al. (1986) (6)
1	F	63	CS	HC	NM	SS and CAS	NM	NM	ST, RT	no	Franscella et al. (1991) (7)
1	F	51	pigmentation, hypertension	HC, EA	NM	SpS and PF	no	no	ST	no	Kamijo et al. (1991) (8)
2	NM	NM	50% (1/2) CS, osteoporosis, hypertension	50% (1/2) HC, 100% (2/2) EA	37	SS and CAS	NM	NM	ST, RT	NM	Ikeda et al. (1997) (9)
1	F	38	CS, hypertension	HC	NM	NM	NM	NM	ST	no	Coire et al. (1997) (10)
1	F	17	HA, secondary amenorrhoea	normal	NM	SS and CAS	yes	sacrum	ST × 4, RT	no	Holthouse et al. (2001) (11)
2	1F, 1M	56.5	50% (1/2) HA, diplopia, tiredness, constipation	50% (1/2) EA, hypopituitarism	NM	SS	no	no	ST	no	Roncaroli et al. (2002) (12)
36	27F, 9M	46	71% (24/34) CS, 76% (13/17) HA, 45% (13/29) VD	94% (15/16) EA, 44% (7/16) HC	NM	67% (18/27) SS	60% (15/25)	8% (2/25)	ST, RT, CT	12% (3/25)	George et al. (2003) (13)
1	F	48	VD, left retro-orbital eye pain	normal	NM	NM	NM	NM	ST	no	Lopez et al. (2004) (14)
1	F	43	CS, VD, HA, fatigue, tiredness	HC, EA	NM	CAS	yes	no	ST × 3	no	Kovacs et al. (2005) (15)
4	4F	44.8	50% (2/4) HA and VD, menstrual irregularities	50% (2/4) HC, hyperprolactinemia	29.8	SS and CAS	25% (1/4)	no	ST, RT	no	Sahli et al. (2006) (16)
2	1F, 1M	51.5	100% (2/2) CS and VD, 50% (1/2) HA	100% (2/2) HC and EA	NM	CAS	100% (2/2)	50% (1/2) spinal	ST, RT, CT	no	Mohammed et al. (2009) (17)
7	5F, 2M	42.9	NM	43% (3/7) HC, 86% (6/7) EA	NM	CAS	57% (4/7)	14% (1/7) liver	ST, RT, CT	no	Takeshita et al. (2009) (18)
1	F	49	CS, VD	HC	15	SS and CAS	yes	no	ST × 4, RT, CT	no	Rotondo et al. (2012) (19)
1	M	52	CS, VD	EA	NM	SS and CAS	yes	no	ST	no	Atkinson et al. (2012) (20)
1	F	16	VD, galactorrhea, amenorrhoea	HC	50	SS	yes	parotid, liver	ST × 8, RT × 3	yes	Kovacs et al. (2013) (21)
1	F	55	CS	HC, EA	30	SS and CAS	yes	no	ST, CT	no	Asimakopoulou et al. (2014) (22)
1	F	58	CS	EA	NM	SS	NM	NM	ST	no	Sathiyabama et al. (2014) (23)
1	M	54	CS	HC, EA	39	SS and CAS	yes	no	ST, CT	no	Kurowska et al. (2016) (24)
1	M	15	delayed puberty	normal	20	no	no	no	ST	no	Glrl et al. (2017) (25)
1	M	61	CS, HA, VD, diabetes mellitus, hypertension	HC, EA	54	SS and CAS	yes	no	ST × 4, RT, CT	yes	Januszewska et al. (2018) (26)
1	F	55	HA, hypertension, hyperlipidemia, dizziness	normal	25	SS	no	no	ST	no	Todnem et al. (2018) (27)
1	M	64	HA, VD, tinnitus	EA	32	CAS	yes	no	ST × 2, RT	no	Khatri et al. (2019) (28)
1	M	45	HA, VD, dizziness, diplopia	EA, hypogonadism	20	SpS, CAS and PF	no	no	ST	no	Randall G et al. (2019) (29)
1	F	56	hypertension, leg edema	HC, EA	57	CAS	yes	no	ST, CT	no	Tanaka et al. (2019) (30)
2	NM	NM	NM	NM	NM	NM	NM	NM	NM	NM	Sema et al. (2019) (31)
1	M	71	HA, impotence, decreased libido	normal	41	SS	no	no	ST, RT, CT	no	Schwann et al. (2020) (32)
1	M	56	post-surgical panhypopituitarism	normal	47	PF	yes	no	ST × 3, RT × 2	no	Cortez et al. (2020) (33)
1	F	39	CS, VD, galactorrhea, hypertension	HC, EA, hyperprolactinemia	30.5	SS and CAS	yes	NM	ST, RT	NM	Ridhi et al. (2021) (34)
5	4F, 1M	46.2	100% (5/5) CS, 60% (3/5) VD	100% (5/5) EA and HC	20.6	SS and CAS	20% (1/5)	no	ST, RT	no	Erica et al. (2021) (35)

CS, Cushing’s syndrome; HA, headache; VD, visual dysfunction; HC, hypercortisolemia; EA, elevated ACTH; SS, suprasellar; CAS, cavernous sinus; SpS, sphenoid sinus; PF, posterior fossa; ST, surgical therapy; RT, radiotherapy; CT, chemotherapy; NM, not mentioned.

We made a retrospective summary of the published cases (**Table 4**) to better understand the clinical features of CCA. Overall, their mean age was 47.8 ± 13.4 years. There were more female patients (72.8%, 59/81) than men (27.2%, 22/81) in CCA patients. The major symptoms in most patients were Cushing’s syndrome (65.1%, 56/86), optic nerve disorder (33.7%, 29/86), and headache (29.1%, 25/86). Notably, up to 71.4% (45/63) of patients were accompanied by an increase in ACTH, while more than half had hypercortisolism. The average diameter of the tumor was 33.2 ± 12.8 mm, and we found that the tumor mainly grew toward the suprasellar (63.8%, 44/69), which coincided with the symptoms of visual impairment in most patients. Additionally, the number of patients with tumor recurrence reached 55.6% (35/63), 6 patients with progression to pituitary carcinoma were reported (9.7%, 6/62), and tumor-related deaths accounted for 7.7% (5/65).

**Table 4:** Characteristics of Reported Literatures.

Classification			Ratio (%)
Gender	Male		27.2 (22/81)
	Female		72.8 (59/81)
Age (y)			47.8 ± 13.4
Clinical manifestation	Cushing’s syndrome		65.1 (56/86)
	Visual defect		33.7 (29/86)
	Headache		29.1 (25/86)
Hormonal dysfunction	Hypercortisolemia		55.6 (35/63)
	Increased ACTH		71.4 (45/63)
Biological characteristics of tumor	Diameter (mm)		33.2 ± 12.8
	Growth direction	Suprasellar	63.8 (44/69)
		Cavernous sinus	46.4 (32/69)
		Sphenoid sinus	2.9 (2/69)
		Posterior fossa	4.3 (3/69)
	Recurrence		55.6 (35/63)
	Metastasis		9.7 (6/62)
	Tumor Related Death		7.7 (5/65)

To better understand the clinical characteristics of CCA, we integrated our cases with the CCA cases previously reported for analysis. As illustrated in **Table 5**, the age composition of the two groups was similar and the data were relatively comparable. Regarding gender, CCA was clearly dominated by female patients. Although this is contrary to the characteristics of our center, it is similar to the overall reporting. In addition, CCA was predominantly a functional tumor with a significantly higher proportion of concomitant Cushing’s syndrome, hypercortisolism and elevated ACTH than non-CCA, supporting the notion that cortisol disorders promote the formation of CCA. The relatively moderate hormone secretion in the non-CCA group resulted in a higher proportion of visual dysfunction than in the CCA group. No significant difference was observed between the two groups with respect to headache. The tumor diameter was significantly larger in CCA than in the control group, but the tendency for tumor invasion into the sphenoid sinus was lower than in non-CCA. In terms of tumor recurrence, the therapeutic effect of CCA was much worse than that of the control group.

**Table 5:** Relationship between tumor type and clinical information.

Categories			CCA	non-CCA	total	χ^2^	t	*p*
Gender		Male	26	17	43	4.284		0.038^*^
		Female	59	80	139			
Age (y)			47.5	45.5			0.7046	0.4823
Clinical Manifestation	Cushing’s Syndrome	yes	58	18	76	40.749		<0.0001^*^
		no	32	79	111			
	Visual Defect	yes	31	49	80	4.926		0.026^*^
		no	59	48	107			
	Headache	yes	28	25	53	0.655		0.418
		no	62	72	134			
Hormonal Dysfunction	Hypercortisolemia	yes	37	8	45	43.923		<0.0001^*^
		no	30	89	119			
	Elevated ACTH	yes	49	18	67	48.851		<0.0001^*^
		no	18	79	97			
Biological Characteristics of Tumor	Suprasellar Extension	yes	47	58	105	0.372		0.542
		no	26	39	65			
	Cavernous Sinus Extension	yes	36	49	85	0.024		0.877
		no	37	48	85			
	Sphenoid Sinus Extension	yes	6	34	40	16.667		<0.0001^*^
		no	67	63	130			
	Posterior Fossa Extension	yes	6	16	22	2.532		0.112
		no	67	81	148			
	Relapsed	yes	38	38	76	4.904		0.027^*^
		no	29	59	88			
	Diameter (mm)		33.9	26.0			2.55	0.0121^*^

*, implies a statistically difference.

## Discussion

Pituitary adenomas are the most common masses of the sellar region arising from adenohypophyseal cells. The pituitary-specific transcription factors, including *PIT-1*, *T-PIT*, *SF-1*, and *GATA-2*, involved in adenohypophysial cell differentiation and maturation are now regarded as key diagnostic tools for the further characterization of pituitary adenomas. Based on the specific transcription factor tested by IHC, the new WHO classification of pituitary tumors has provided an integrated approach for the diagnosis and classification of pituitary adenomas (36). *T-PIT*-driving corticotroph adenomas represent 10 to 15 percent of all pituitary adenomas and are divided into three recognized variants: densely granulated adenomas, sparsely granulated adenomas, and Crooke’s cell adenomas. Crooke’s cell adenoma is an uncommon variant of *ACTH*-immunoreactive adenoma in which the cells recapitulate Crooke’s hyaline change observed in the non-neoplastic pituitary gland under the influence of elevated cortisol levels. In 1935, Crooke et al. first reported the hyaline change of basophils in the anterior pituitary (2). Subsequent studies found intermediate filament-rich Crooke’s cells, with ring-like cytoplasmic filaments accumulating, causing dispersal of secretory granules (and both PAS and *ACTH* reactivity) to the peripheral submembrane region or displaced internally next to the nucleus. Porcile and Racodot (37) confirmed that the core material of the hyalinization was made up of 7nm filaments arranged in parallel circles surrounding the nucleus by the ultrastructural method. The remaining secretory granules and other organelles were divided into perinuclear and perimembrane groups. Subsequently, Newman et al. (38) revealed that Crook’s hyaline deposition was immunohistochemically composed of intermediate filament keratin. Yet, until now, there is no clear mechanism to explain the phenomenon of increased keratin. Some studies suggest that Crooke-like hyalinization is a cellular inhibitory response to cortisol stimulation, such as the fact that Crooke’s cells are commonly accompanied by hypercortisolemia, and the phenomenon that organelles like the Golgi apparatus are squeezed around the cell membrane is considered a manifestation of inhibition of cell function (5, 9). Our results also confirm the above point by analyzing about 200 cases (**Table 5**). Irregular processing of precorticotropin (*POMC*), a precursor of corticotropin, is thought to be the cause of clinical silence in these tumors (27). Crooke-like change is generally a change in the response of normal corticotropin cells, which is not often involved in *ACTH* tumor cells. This is due to the expression of the glucocorticoid receptors in normal corticotropin cells, while the low or no expression of the glucocorticoid receptors in tumor cells leads to their tolerance to hypercortisolemia (12). Studies have confirmed that the keratin filaments accumulated in Crooke-like *ACTH* cells are *CK8* and *CK18* (39). Some studies have shown that *CK20* is also involved in the formation of Crooke-like deposition. According to 2022 WHO classification guidelines (36) and previous case reports, *CK8/18* immunostaining is described as a “perinuclear and ring-shaped” change. In 2003, George et al. (13) published a study with the largest number of cases to date. They took the positive rate of *CK8/18* circular immunostaining > 50% as a diagnostic criterion for CCAs, which is still in use today.

Pathological diagnosis of CCA depends on the results of typical histology and immunostaining. In a typical Crooke’s cell adenoma, more than 50% of tumor cells exhibit obvious intracytoplasmic circular hyalines stained by HE and *CK8/18* immunostaining, lack of intact reticulin scaffold, and positive *T-PIT* immunostaining (13). In addition, there is an eccentric rhabdoid keratin staining with neoplastic Crooke’s cells, although circular enhancement is the earliest and most characteristic change (21). Our medical records also show that semi-circular and strip-shaped keratin staining co-exists with circular enhancement in variable proportions. We note that the circular characteristic of *CK8/18* staining is not the only way of expression, and the types of semi-cyclic and strip staining should attract the attention of researchers as well. It is not an unusual phenomenon that our cases contain various semi-ringlike *CAM 5.2* positive cells (**Figures 1D**, **3D**, **5D**, **7D**). The *Ki-67* is even as high as 40% in case1, reminding us that despite the existence of atypical Crooke-like cells, the invasiveness of the tumor still needs the close attention of researchers. In addition, the proportion of Crooke-like positive cells is worthy of further study. Conversion of adrenocorticotropic hormone cells to Crooke’s cells is reported to account for up to 25% of 52% of *ACTH* adenomas (40). Another study found that in 177 patients with neuroendocrine tumors with positive corticotropin staining confirmed by histology and 213 patients with Cushing’s syndrome diagnosed by pituitary surgery, about 74% of the tumor samples experienced Crooke-like changes (41). Also, it has been suggested that as long as the anterior lobe biopsy measures more than 25% of Crook’s hyaline deposition, it may indicate that the functional recovery of the HPA axis is slower after the operation (25, 42). A recent study pointed out that due to local infiltration and growth, non-tumor tissue is very common in *ACTH* tumor specimens, and how to identify normal tissue in pathological diagnosis is very important (39). It is not known whether different percentages of Crooke’s cells indirectly indicate the degree of tumor invasion, but it can be predicted that once Crooke-like hyaline change occurs in tumor tissues, its invasive behavior is also stronger than that of general *ACTH* adenomas (9, 43), which deserves the continuous attention of clinicians. Combined with previous literature reports, considering the formation of Crooke’s cells is a long-term outcome of hypercortisolemia, which makes us wonder: is “50%” in the previous diagnostic criteria appropriate? After all, Crooke’s cell tumor is a highly invasive tumor, and its early diagnosis has a positive sign for the clinical prognosis of the disease.

Treatment of CCAs remains challenging due to its aggressive nature and high recurrence rate. According to the case summaries in our center and the literatures, the diameter of the CCAs is significantly larger than that of non-CCAs’ (**Tables 1** and **5**). Unlike the proportion reported, all CCAs from our center were male (**Table 1**). In general, the surgical treatment goal of CCAs is still gross-total resection. However, despite the effectiveness of surgical resection, its recurrence rate remains as high as 56.7% (38/67) and the success rate of reoperation is low: case 1 died of severe complications despite multiple treatments, while global tumor-related mortality reached 8.7% (6/69). Radiotherapy may be considered for patients with postoperative recurrence, postoperative residues, or strong invasiveness. A recent study (44) reported that 40% of the patients who received radiotherapy had a 30% reduction in tumor volume, and there was no tumor recurrence or growth during the 12-month follow-up, no complications of radiotherapy, and no patients experienced a second dose of radiotherapy. In this study, radiotherapy is used as an adjuvant for surgery rather than as an independent treatment, since all patients have received at least one surgical treatment. Temozolomide (TMZ), a first-line drug against glioma, is also effective in treating refractory pituitary adenomas. One study revealed that the overall effective rate of TMZ in the treatment of refractory pituitary adenomas was 45%, and 27% of people were in stable condition (26). *ACTH* adenomas, particularly invasive tumors, have a low level of *MGMT* (18), which is an indication for TMZ treatment. However, some studies have shown that as the malignant degree of CCA increases, its *MGMT* level increases, and the effectiveness of TMZ treatment decreases. The effectiveness of TMZ has received positive signals in clinical practice. At least 18-33 months (22, 24) after discontinuation of TMZ treatment, the disease is still in remission, and these data demonstrate the safety and effectiveness of discontinuing temozolomide treatment. A recent study confirmed that no tumor recurrence was found in the seven years after TMZ treatment (30). In addition to using the level of *MGMT* as a reference for tumor treatment, genetic mutations and small molecule RNA also provide potential entry points for tumor treatment. Kyohei Hayashi et al. (45) reviewed 60 cases of *ACTH* adenoma (including 15 cases of Crooke’s cell adenoma). They found that the *USP8* mutation rate was high and the downstream *POMC* content was also increased in microadenomas, while macroadenomas such as CCAs had a low *USP8* mutation rate accompanied by decreased expression of *POMC* and *MGMT*, suggesting the suitability of CCAs for TMZ therapy. Garbicz et al. (46) found that *miR-106b-25* and its host gene *MCM7* are potential novel biomarkers of invasive corticotropin immunopositive pituitary adenomas. We have a number of *T-PIT* cases in our NFPAs, and the previously reported anti-oncogene *MEG3* (47) may also serve as a breakthrough in treatment. The percentage of *T-PIT*-positive in NFPAs reported in our center is close to the results of a Japanese study (26.9%) (48), but higher than the prevalence reported in previous literature (5.0-19.0%) (49). The reasons for this result were considered to be caution in diagnoses making and a decrease in the number of asymptomatic patients seeking medical care.

## Conclusion

CCA is a rare pituitary adenoma that has received significant attention because of its aggressive nature and high recurrence rate. It should be assisted by a multidisciplinary consultation to deal with this particular type of tumor from the initial stages rather than once the recurrence has already occurred. However, the percentage of tumors immunopositive for keratin and their presentation status are confronted with clinical diversity, and very few studies involving the exploration of their pathogenesis have been performed. Therefore, more cases need to be investigated to further reveal the clinical features of CCA and its underlying mechanisms.

## Data availability statement

The original contributions presented in the study are included in the article/**Supplementary Material**. Further inquiries can be directed to the corresponding authors.

## Ethics statement

The studies involving human participants were reviewed and approved by Medical Ethics Committee of the First Affiliated Hospital of Sun Yat-sen University. The patients/participants provided their written informed consent to participate in this study.

## Author contributions

DZ: literature review and draft writing; ZW: clinical data analysis; TT and XW: clinical data collection; DH and YZ: manuscript revision; DL and HW: pathological sections analysis and research design. All authors contributed to the article and approved the submitted version.

## Funding

Our research was supported by the Guangzhou Science and Technology Project (grant no. 202102021116) and Sun Yat-sen University Clinical Research 5010 Program (grant no. 2016008).

## Acknowledgments

Thank the members of Prof. Yonghong Zhu’s team for their help during the implementation of the study.

## Conflict of interest

The authors declare that the research was conducted in the absence of any commercial or financial relationships that could be construed as a potential conflict of interest.

## Publisher’s note

All claims expressed in this article are solely those of the authors and do not necessarily represent those of their affiliated organizations, or those of the publisher, the editors and the reviewers. Any product that may be evaluated in this article, or claim that may be made by its manufacturer, is not guaranteed or endorsed by the publisher.
