# Supplementary material for: Prevalence and clinical characteristics of Crooke’s cell adenomas in 101 patients with T-PIT-positive pituitary adenomas: Case series and literature review
Source: Front Endocrinol (Lausanne). 2022 Aug 19;13:947085. doi: 10.3389/fendo.2022.947085 (PMC9437488; doi:10.3389/fendo.2022.947085)
Supplement: Supplementary file 1 [file Table_1.docx]

Supplemental Table 1. The overlap with clinical and hormone immunohistochemistry

| IHC Clinical hormone | ACTH (+) | PRL (+) | GH (+) | TSH (+) | FSH (+) | LH (+) |  |
| --- | --- | --- | --- | --- | --- | --- | --- |
|  |  |  |  |  |  |  |  |
| Elevated ACTH | 22 | 2 | 2 | 2 | 2 | 4 |  |
| Normal ACTH | 68 | 8 | 8 | 5 | 5 | 22 |  |
| Total | 90 | 10 | 10 | 7 | 7 | 26 |  |

IHC, immunohistochemistry.
